# Nonlinear Characteristics of Tensile and Compressive Mechanical Parameters for Typical Pavement Materials

**DOI:** 10.3390/ma19030536

**Published:** 2026-01-29

**Authors:** Qinxue Pan, Yuting Tan, Jia Hu, Long Zhang, Songtao Lv, Haihui Duan, Pan Tan, Jiang Yuan, Tongsheng Zhang, Xiaojin Song, Xudong Zha

**Affiliations:** 1 School of Transportation, Changsha University of Science and Technology, Changsha 410114, China; 15272854779@163.com (L.Z.); lst@csust.edu.cn (S.L.); hhduan@csust.edu.cn (H.D.); tanpan@csust.edu.cn (P.T.); jyuan@tongji.edu.cn (J.Y.); zhaxd410@163.com (X.Z.); 2 National Key Laboratory of Green and Long-Life Road Engineering in Extreme Environment, Changsha 410114, China; 3 National Engineering Research Center of Highway Maintenance Technology, Changsha 410114, China; 4 Chengnan College, Changsha University of Science & Technology, Changsha 410015, China; 15973107992@163.com; 5 Shandong Hi-Speed Transportation Construction Group Co., Ltd., Jinan 250101, China; 17862351819@163.com; 6 Zhongteng Zhixin Technology (Hunan) Co., Ltd., Changsha 410036, China; sxjmail@126.com

**Keywords:** pavement materials, mechanical parameters, difference in tension and compression, gradation, quantitative model

## Abstract

The reliability of the mechanical property values adopted for current pavement structural design remains low. Therefore, this study investigated the nonlinear characteristics for the tensile and compressive properties of cement-stabilized macadam (CSM) and an asphalt mixture (AM) under different gradation types and loading rates. And a multi-factor value model was developed for both. The results show that the tensile and compressive stress–strain behavior of both CSM and AM exhibited bilinear characteristics consistent with the bi-modulus theory (elasticity with different moduli in tension and compression). The strength, elastic modulus, and Poisson’s ratio followed a power function relationship with increasing loading rates, stabilizing at values beyond 0.1 MPa/s. The skeleton-dense gradation demonstrates the most favorable mechanical performance. For semi-open-graded AM with a void ratio of 8~16%, the mechanical parameters exhibit relatively high rates of change. Among the influencing factors, gradation type had the most significant impact on the mechanical parameters, especially on the elastic modulus. In general, tensile mechanical parameters were more sensitive to changes than compressive ones. *R*_c_/*R*_t_ was most strongly affected by gradation type. Accordingly, a quantitative value model was established to describe the variation in tensile and compressive mechanical parameters of typical asphalt pavement materials, which vary with air void ratio, loading rate, binder content, and temperature. The findings provide a reference for the prediction of pavement structure design parameters considering the difference in compression and tension.

## 1. Introduction

Most pavement materials have significant differences in tension and compression [1,2]. However, the current pavement structural design in China still adopts the compressive elastic modulus as the mechanical input for structural analysis. This may cause discrepancies between predicted results and actual mechanical responses. At the same time, pavement structure calculation and design results are significantly affected by the value of mechanical parameters [3,4,5]. However, the material mechanical parameters in current pavement structure design specifications are generally selected by experience or reference-recommended ranges. The quantitative influence of factors such as the internal composition of the material and the external environment and the correlation between the mechanical parameters are not reasonably considered. This causes the material’s mechanical parameters and its real mechanical properties to have significant differences. Therefore, it is of great significance to study the variation characteristics and value models of mechanical parameters of typical asphalt pavement materials under the influence of multiple factors.

Gradation, as a key parameter in pavement material mix design, fundamentally governs aggregate interlock and the formation of the internal void structure. Consequently, it plays a decisive role in determining strength, modulus, and other essential mechanical properties and serves as a critical basis for ensuring structural bearing capacity and long-term performance of pavement systems. For asphalt mixtures, optimized gradation is required to achieve coordinated action between the aggregate skeleton and asphalt film, thereby improving high-temperature rutting resistance while maintaining adequate low-temperature fatigue cracking resistance [6,7]. In contrast, the gradation design of cement-stabilized macadam focuses more on enhancing structural stiffness and resistance to shrinkage cracking in the base layer [8]. The compatibility between the mechanical properties of these two material systems directly affects interlayer stress transfer and, ultimately, the service life of the pavement structure. Under current engineering conditions characterized by increasingly heavy traffic and frequent extreme weather events, conventional gradation designs may result in a mismatch between mechanical performance and service demands, leading to premature distresses such as rutting and reflective cracking. Therefore, systematically investigating the influence of different gradation types on the mechanical properties of pavement materials is essential for supporting mix design refinement and improving the reliability of parameter selection for pavement structural analysis. Mansour et al. [9,10] found that mechanical parameters such as the indirect tensile strength, splitting strength, and elastic modulus of AM can vary by orders of magnitude with changes in aggregate gradation. Zhou et al. [11] showed experimentally that CSM with a skeleton-dense gradation exhibits higher 28-day flexural strength, 28-day compressive strength, and absolute fatigue strength than that with a skeleton-void structure. Additionally, Jiang et al. [12,13,14,15] investigated the variation patterns of the mechanical properties of pavement materials in response to factors such as aggregate content and loading rate. Some researchers [16,17] have developed relationship models for pavement mechanical properties under single or partially multiple influencing factors based on simplified quantitative analyses. However, these models often exhibit limited accuracy or fail to comprehensively account for the combined effects of various influencing factors. Therefore, it is necessary to investigate the variation in mechanical parameters under the combined effects of multiple factors (e.g., gradation type, loading rate, temperature, and binder content) to establish an accurate quantitative model.

In the 1940s, the former Soviet scholar Timoshenko [18] first introduced the concept of bi-modulus materials. Later, in 1982, Ambartsumyan [19] further advanced this field by publishing a monograph on the theory of different elastic moduli, in which he derived the generalized elastic law of bi-modulus theory and simplified the stress–strain relationship of materials with different moduli into a bilinear model, thereby establishing the theoretical foundation for bi-modular mechanics. Lv et al. [20,21,22] experimentally demonstrated that pavement materials exhibit significant differences in tension and compression. Their findings indicate that using a single compressive modulus for structural analysis leads to substantial discrepancies between calculated results and actual mechanical responses. Studies have shown that, once the tensile–compressive modulus difference is considered, the plate response can differ by more than 20% from predictions based on linear elasticity with a single (equal) modulus. [23] Wu et al. [24] examined large-deflection bending of bi-modulus thin plates. They showed that when the tensile and compressive elastic moduli differ substantially, the equal-modulus theory is not suitable for calculating plate deflection. Zhang et al. [25] argued that using a single modulus in conventional rigid pavement designs is unreasonable. They derived bi-modulus-based design equations using the Winkler foundation model. They found that the maximum tensile stress at the critical load location is reduced by about 10% compared with the traditional method. In recent years, Pan et al. [26] proposed a three-dimensional numerical method for asphalt pavements. The method uses an iterative scheme and a bi-modulus constitutive relationship for asphalt mixtures. They reported the largest differences between bi-modulus theory and conventional linear-elastic theory. The maximum discrepancies were about 50% for rigid-base pavements, 55% for inverted structures, and 33% for flexible-base pavements. Therefore, it is important to consider the different mechanical properties in tension and compression in pavement materials. This is essential for developing more refined asphalt pavement design methods.

In summary, the analysis of asphalt pavements based on the bi-modulus theory still lacks a solid basis for selecting tensile and compressive mechanical parameters. To address this issue, this study examines two representative pavement materials: cement-stabilized macadam and asphalt mixtures. Uniaxial compression tests and direct tensile tests were conducted under different gradation types and loading rates. The results reveal how compressive and tensile strength, elastic modulus, and Poisson’s ratio vary with gradation type and loading rate. Differences between tensile and compressive parameters are then compared and discussed. The fundamental assumptions of bi-modulus theory are also examined based on these results. Finally, a multi-factor parameterization model is developed for typical pavement materials. It accounts for gradation type, loading rate, temperature, and binder content. The model is intended to support rational selection of tensile and compressive parameters in asphalt pavement structural design based on the bi-modulus theory.

## 2. Test Scheme

### 2.1. Raw Materials and Proportioning Design

#### 2.1.1. Raw Materials

The P.C. 32.5 cement produced by Hunan Southern Cement Co. (Changsha, China), SBS (I-D)-modified asphalt produced by Hunan Baoli Asphalt Co. (Changsha, China), and basalt aggregate produced by Hunan Yunzhong Technology Co. (Changsha, China) were used in the test. The test showed that all its indexes met the requirements of JTG 3420-2020 [27], JTG 3410-2025 [28], and JTG 3432-2024 [29]. The test results are shown in Table 1, Table 2 and Table 3.

#### 2.1.2. Void Ratio Test and Gradation Design

**Figure 1:**
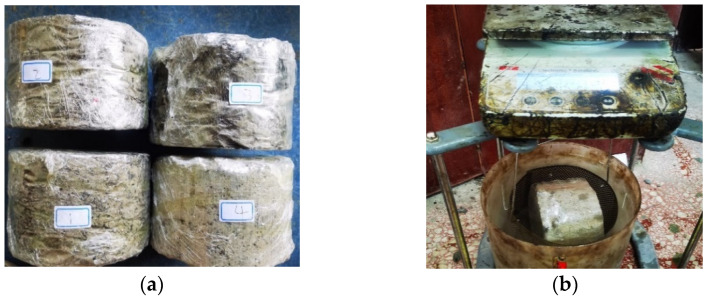
Void ratio test of CSM. (**a**) Film seal specimen. (**b**) Void ratio test.

Combined with engineering practice and referring to the technical construction specifications, four gradations of CSM (covering suspension-dense, skeleton-dense, and skeleton-void types) and five gradations of AM (including dense-graded, semi-open-graded, and open-graded types) were designed. This study referred to JTG 3410-2025 and used the film-sealing method to test the void ratio of both materials. The testing process is shown in Figure 1.

The gradation designs for CSM and AM are presented in Table 4 and Table 5. The optimum moisture content and maximum dry density for CSM are detailed in Table 6, while the results of the Marshall test for AM are summarized in Table 7.

#### 2.1.3. Specimen Forming and Curing

The uniaxial compressive specimens for CSM were cylindrical, with a height of 150 mm and a diameter of 150 mm. In contrast, the direct tensile specimens were beam-shaped, measuring 400 mm × 100 mm × 100 mm. After preparation, the specimens were cured under standard conditions (temperature: 20 ± 2 °C, humidity ≥ 95%) for 90 days. The uniaxial compressive specimens for AM were also cylindrical, with a height of 100 ± 2 mm and a diameter of 100 ± 2 mm. The direct tensile specimens were prepared by cutting a 300 mm × 300 mm × 50 mm rutting plate into five beam-shaped specimens, each measuring 250 mm × 50 mm × 50 mm for testing.

### 2.2. Mechanical Parameters’ Test Scheme

#### 2.2.1. Test Scheme

To study the nonlinear characteristics of compressive and tensile mechanical properties of typical pavement materials under different void ratios and loading rates, the cement dosage for CSM was 5%, the asphalt content for AM was 4.5%, and the test temperature for AM was 15 °C. Previous studies have indicated that the air void content of porous CSM used in drainage base layers and permeable AM is generally around 20%, while that of dense-graded asphalt concrete (AC) is typically about 4%, and that of suspension-dense CSM is approximately 6%. Accordingly, considering practical construction and laboratory conditions, representative gradation types were selected, and a series of tests was carried out under different loading rates (0.01, 0.05, 0.1, and 0.5 MPa/s) to examine the variation characteristics of the tensile and compressive mechanical parameters of these typical pavement materials.

#### 2.2.2. Test Method

**Figure 2:**
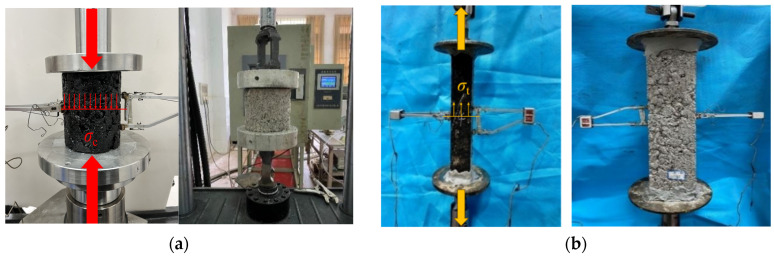
Uniaxial compressive and direct tensile test. (**a**) Uniaxial compressive test. (**b**) Direct tensile test.

This study investigated the relationship between the void ratio and the mechanical properties of CSM and AM through uniaxial compressive and direct tensile tests. These experiments were conducted in accordance with the Chinese standards JTG 3441-2024 [30] and JTG 3410-2025. An external extensometer was employed to measure longitudinal and transverse strains for the determination of modulus and Poisson’s ratio. The stress state of the specimens during testing is illustrated in Figure 2.

During testing, the peak failure load was recorded, and the uniaxial compressive and tensile strength were calculated using the following formula:

(1) $$R_{c}=\frac{4P_{c}}{\pi d^{2}}$$

(2) $$R_{t}=\frac{P_{t}}{bh}$$

where *R*_c_ and *R*_t_ are compressive and tensile strength, respectively (MPa). *P*_c_ and *P*_t_ are the peak loads corresponding to the destruction of the specimen in compression and tension tests, respectively (N). *d* is the diameter of the cylinder specimen (mm). *b* and *h* are the side lengths of the failure section of the beam specimen (mm).

The specimens were subjected to a stepwise loading and unloading process. A five-stage loading and unloading procedure was implemented for CSM, while a seven-stage procedure was adopted for AM. Each stage applied loads ranging from 0.1 *P_m_* to 0.7 *P_m_* (in 0.1 *P_m_* increments, where *P_m_* represents the maximum failure load), with a 30 s pause after each unloading before proceeding to the next loading stage. Longitudinal and transverse strains during the loading and unloading phases were measured using extensometers, and the modulus was calculated using the following formula.

(3) $$E_{c}=\frac{4{P_{c}}^{\prime }}{\pi d^{2}\varepsilon_{c}}$$

(4) $$E_{t}=\frac{{P_{t}}^{\prime }}{bh\varepsilon_{t}}$$

where *E*_c_ and *E*_t_ are the compressive and tensile modulus of the specimen, respectively (MPa). *P_c_*′ and *P_t_*′ are compressive and tensile loads per stage (N). *ε_c_* and *ε*_t_ are uniaxial compressive and direct tensile vertical rebound strains, respectively.

The Poisson’s ratio was calculated using the following formula, which is based on the longitudinal and transverse strains measured by extensometers.

(5) $$\mu =\left|\frac{\varepsilon_{h}}{\varepsilon_{v}}\right|$$

where *μ* is the Poisson’s ratio of the specimen. *ε*_h_ and ε_v_ are the transverse and longitudinal strains of the specimen, respectively.

**Figure 3:**
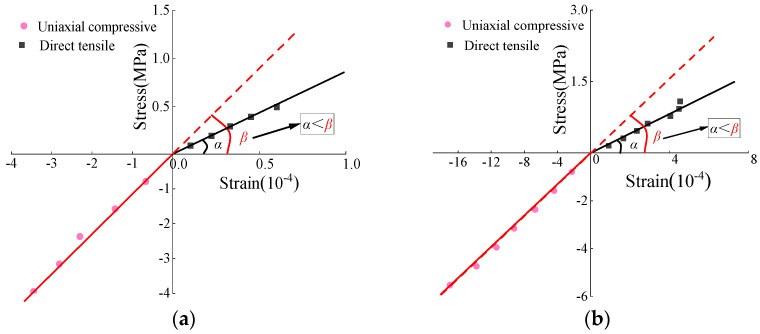
Tensile and compressive stress–strain bilinear model. (**a**) CSM. (**b**) AM.

The measured stress–strain curves of CSM and AM under various tensile and compressive load levels are presented in Figure 3. The results indicate that both materials exhibit distinct bilinear characteristics in tension and compression, consistent with the stress–strain behavior predicted by bi-modulus theory. Similar patterns are observed when considering a combination of various influencing factors. In comparison to the direct tensile stress state, the stress–strain curves under uniaxial compression demonstrate steeper slopes, and the angle between the curves and the horizontal axis is greater, resulting in a higher uniaxial compressive modulus than the direct tensile modulus.

## 3. Study on the Nonlinear Relationship of Compressive and Tensile Mechanical Parameters of Asphalt Pavement Materials

### 3.1. Analysis of Mechanical Parameter Test Results of CSM

#### 3.1.1. Strength

**Figure 4:**
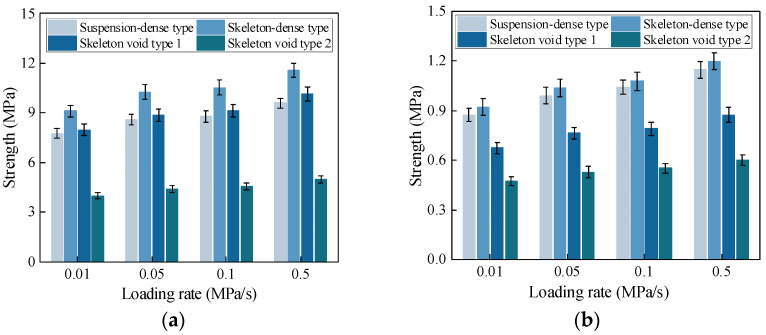
Strength of CSM. (**a**) Uniaxial compressive strength. (**b**) Direct tensile strength.

The variation in compressive and tensile strengths with loading rates for CSM with different gradation types is shown in Figure 4.

The variation in strength with loading rate for CSM with different gradation types is shown in Figure 4. It can be seen that the strength first increases and then decreases as the air void content increases. This trend is strongly related to the gradation type. However, the effect of gradation on tensile and compressive strength is not the same. In the compression test, the strengths of skeleton-void type 1 and the suspension-dense CSM are similar. In the direct tension test, the tensile strength of the skeleton-void type 1 material is much lower than that of the suspension-dense material. The skeleton-dense CSM shows the highest tensile and compressive strengths. The maximum differences in compressive and tensile strength between different gradation types are about 95% and 130%, respectively.

This behavior is controlled by the different load-resisting mechanisms in tension and compression. Under compression, the material mainly relies on aggregate interlock and the strength of the aggregate itself. A proper increase in coarse aggregate content can raise the strength. When the air void content increases, the strength decreases. At low to moderate void contents, the compressive strength changes only slightly. When the air void content reaches about 20%, the internal interlock is greatly reduced, and the compressive strength drops sharply. Under tension, the aggregate strength has a smaller effect on the mixture strength. The load is mainly carried by the bond between the aggregates and the cement paste. As a result, the tensile strength is less sensitive to air void content than the compressive strength. When the air void content is between 10% and 20%, the tensile strength is approximately linearly related to the void content.

The strength of CSM increases with loading rate, but the growth in tensile and compressive strength becomes smaller and tends to level off. Taking the suspension-dense CSM as an example, when the loading rate increases from 0.01 MPa/s to 0.1 MPa/s, the compressive and tensile strengths increase by about 14% and 17%, respectively. When the loading rate increases further from 0.1 MPa/s to 0.5 MPa/s, the compressive and tensile strengths increase by only about 9% and 11%, respectively. Thus, at higher loading rates, the strength of CSM is less sensitive to loading rate.

#### 3.1.2. Modulus

**Figure 5:**
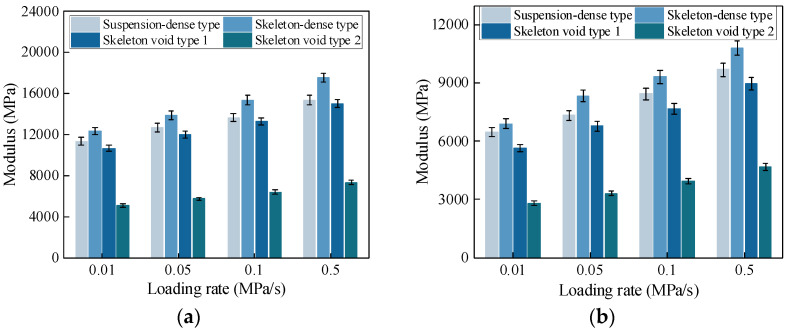
Modulus of CSM. (**a**) Uniaxial compressive modulus. (**b**) Direct tensile modulus.

Based on the test results, the variation in compressive and tensile moduli with loading rates for CSM with different gradation types is shown in Figure 5.

The variation in compressive and tensile modulus with loading rates for CSM with different gradation types is shown in Figure 5. Both the compressive and tensile modulus increase first and then decrease as the air void content increases. The influence of gradation type is significant. Among the three gradation types, the skeleton-dense CSM shows the highest modulus, while the skeleton-void CSM shows the lowest. The maximum differences in compressive and tensile modulus between the skeleton-dense and skeleton-void CSM are approximately 140% and 120%, respectively. This behavior is attributed to differences in the internal load-carrying mechanisms. At lower air void contents, deformation resistance mainly depends on the bonding between cement paste and aggregates. At higher void contents, the contact area between coarse aggregates becomes smaller, leading to greater deformation. For the skeleton-dense gradation, the higher coarse aggregate content improves the internal structure and provides better resistance to deformation. Therefore, using skeleton-dense gradation in semi-rigid base pavement is beneficial for improving deformation resistance.

The modulus increases with loading rate; however, the rate of increase gradually decreases. In addition, the sensitivity of both tensile and compressive modulus to loading rate becomes more pronounced as the air void content increases.

#### 3.1.3. Poisson’s Ratio

**Figure 6:**
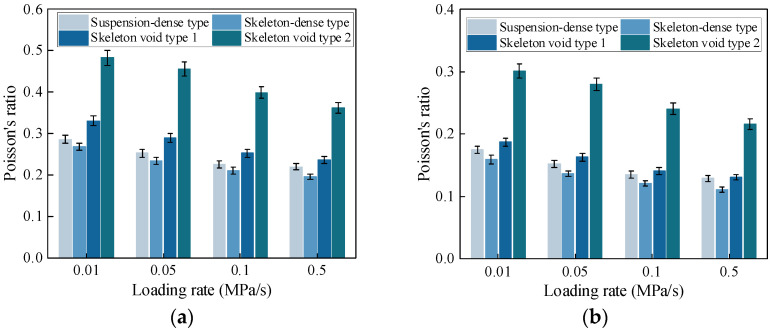
Poisson’s ratio of CSM. (**a**) Uniaxial compressive Poisson’s ratio. (**b**) Direct tensile Poisson’s ratio.

Based on the test results, the variation in compressive and tensile Poisson’s ratios with loading rates for CSM with different gradation types is shown in Figure 6.

The variation in Poisson’s ratio for CSM with different gradation types is shown in Figure 6. The skeleton-dense CSM has the lowest compressive and tensile Poisson’s ratios, while the skeleton-void CSM has the highest. This is because the bonding between cement paste and aggregates is better in the dense skeleton structure. In addition, when the overall density does not change much, an increase in coarse aggregate content can reduce lateral deformation, which leads to a lower Poisson’s ratio for the skeleton-dense CSM.

The compressive and tensile Poisson’s ratios decrease as the loading rate increases. When the loading rate is below 0.1 MPa/s, Poisson’s ratio is more sensitive to changes in loading rate. At higher loading rates, it tends to be a nearly constant value. At a fixed air void content, when the loading rate increases from 0.01 MPa/s to 0.5 MPa/s, the compressive and tensile Poisson’s ratios decrease by about 25% and 30%, respectively.

### 3.2. Analysis of Mechanical Parameter Test Results of AM

#### 3.2.1. Strength

**Figure 7:**
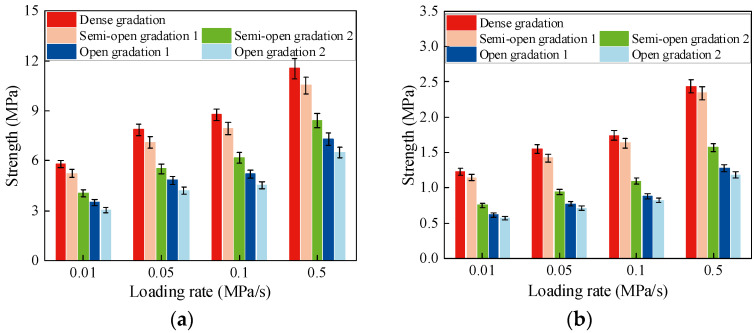
Strength of AM. (**a**) Uniaxial compressive strength. (**b**) Direct tensile strength.

Based on the test results, the variation in compressive and tensile strengths with loading rates for AM with different gradation types is shown in Figure 7.

The influence of gradation type on the tensile and compressive strength of AM is shown in Figure 7. When the air void content increases to 16%, both tensile and compressive strengths decrease, but the reduction in tensile strength is smaller than that in compressive strength and gradually tends to a stable value. This behavior is mainly governed by different load-resisting mechanisms. As the air void content increases, aggregate interlock is weakened, so the compressive strength continues to decrease. In tension, the AM mainly resists load through the bond between the asphalt binder and aggregates. When the air void content approaches about 20%, the reduction in fine aggregates has a limited effect on strength, and further increases in air voids cause only a slight decrease in tensile strength, which tends to be at a nearly constant level. These results also confirm the role of fine aggregates in AM. For dense-graded and semi-open-graded AM, the filling effect of fine aggregates enhances both tensile and compressive strength, and strength is positively correlated with the fine aggregate content. For high-void AM, the influence of fine aggregate content on mechanical strength is much smaller, so gap-graded designs can be adopted for these mixtures.

As shown in Figure 7, the tensile and compressive strengths of AM with different gradation types vary in loading rate in the same way as those of CSM but with a larger increase in magnitude. At low loading rates, failure is mainly governed by cohesive cracking within the asphalt mastic and at the mastic–aggregate interface. As the loading rate increases, more load is carried by the coarse aggregates, and the dominant failure mode shifts toward aggregate fracture. This leads to an increase in strength with loading rates. However, because the strength of the coarse aggregates is limited, the increase in mixture strength becomes smaller at higher loading rates and eventually approaches a plateau.

#### 3.2.2. Modulus

**Figure 8:**
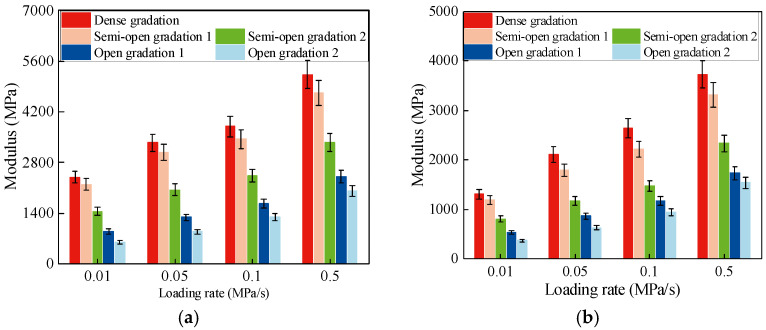
Modulus of AM. (**a**) Uniaxial compressive modulus. (**b**) Direct tensile modulus.

Based on the test results, the variation in compressive and tensile moduli with loading rates for AM with different gradation types is shown in Figure 8.

The modulus of the dense-graded AC is the highest, and that of the open-graded AM is the lowest, as shown in Figure 8. The tensile and compressive modulus of AM both decrease as the air void content increases. The tensile and compressive modulus of the dense-graded AC are about 3.8 times and 3.4 times those of the open-graded AM, respectively. When the air void content is between 4% and 8%, the modulus decreases slowly. When the air void content is between 8% and 16%, the modulus drops sharply with further increases in air voids. When the air void content exceeds 16%, the decreasing trend becomes less pronounced. The reason for this is similar to that for strength. The ability of the mixture to resist deformation decreases as the void content increases. When the air void content is greater than about 20%, deformation is mainly resisted by the cohesion between the aggregates and binder, so the deformation and modulus tend to stabilize.

For all gradation types, the modulus of AM shows essentially the same loading-rate trend as cement-stabilized macadam, but the increase is about 30% higher. The mechanism is similar to that of strength. The internal deformation-resisting mechanism changes with loading rate. At low loading rates, deformation is mainly resisted by the cohesive forces within the AM. At high loading rates, in addition to cohesion, the aggregate skeleton also contributes to deformation resistance because the instantaneous stress is higher. As a result, deformation decreases with increasing loading rates, and the modulus increases accordingly.

#### 3.2.3. Poisson’s Ratio

**Figure 9:**
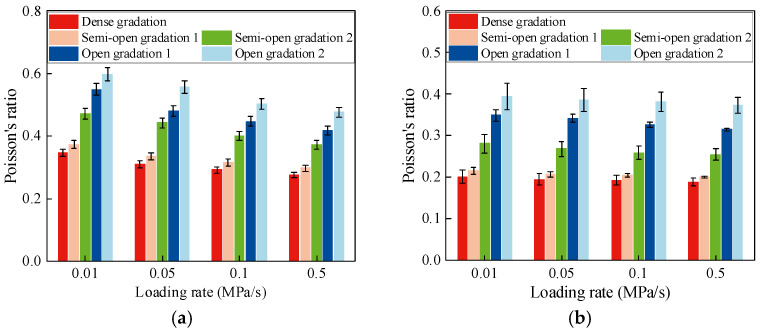
Poisson’s ratio of AM. (**a**) Uniaxial compressive Poisson’s ratio. (**b**) Direct tensile Poisson’s ratio.

Based on the test results, the variation in compressive and tensile Poisson’s ratios with loading rates for AM with different gradation types is shown in Figure 9.

Figure 9 shows that the compressive and tensile Poisson’s ratios of the AM both increase with air void content. When the air void content is between 4% and 8%, Poisson’s ratio increases slowly. In the range of 8~16%, the growth rate increases significantly. When the air void content exceeds 16%, the increase in Poisson’s ratio becomes less pronounced. At an air void content of 19.7% (open-graded AM), the compressive Poisson’s ratio approaches or even exceeds 0.5. This occurs because the fine aggregate content is greatly reduced, the lateral deformation increases sharply, and obvious plastic deformation may develop, leading to unrealistic Poisson’s ratio values. Thus, at high void contents, the elastic behavior of the AM is weakened, and the response is dominated by plastic deformation.

The variation in compressive and tensile Poisson’s ratios for AM with loading rates is basically consistent with that of CSM. However, within the range of loading rates considered, the reduction in compressive and tensile Poisson’s ratios in AM is about 5% and 23% lower than that in CSM, respectively.

### 3.3. Typical Asphalt Pavement Material Value-Taking Model Under the Influence of Multiple Factors

In previous experimental studies on the mechanical properties of materials conducted by the author’s research team [31,32,33,34,35], the effects of temperature (5 °C, 15 °C, 20 °C, 30 °C, and 40 °C), cement content (1%, 2%, 3%, 4%, and 5%), and asphalt content (3.5%, 4%, 4.5%, 5%, and 5.5%) were examined. Tests were performed to assess the compressive and tensile strengths, elastic moduli, and Poisson’s ratios of CSM and AM under these varying conditions. The research findings revealed the following:

For CSM, as the cement dosage increases, the initial hydration products are less generated, resulting in a slow increase in strength and modulus. When the cement dosage is moderate, the hydration products fully fill the pores and form a skeleton, leading to a significant performance increase. However, when the cement dosage exceeds the saturation point, due to the constraints of the aggregate skeleton and the limitations of volume stability, the performance growth tends to plateau and approach the limit value. Therefore, the relationship between its compressive strength, modulus, and cement dosage can be characterized using the Logistic function and the S-shaped logistic function, respectively. The compressive Poisson’s ratio decreases with increasing cement dosage, indicating that increasing the cement dosage effectively enhances the mixture’s ability to constrain lateral deformation; the two exhibit a quadratic function relationship.

For AM, both their compressive and tensile strengths and moduli decrease with increasing temperatures. This trend can be characterized using an exponential function, i.e., a rapid initial decrease followed by a gradual plateau and approaching a stable value. This model agrees well with existing experimental data. When the asphalt content is low, the bond strength between the aggregate and the asphalt is insufficient, resulting in lower strength and modulus. As the asphalt content increases, the bonding performance improves, and the mechanical properties gradually increase. However, when the asphalt content is excessive, the amount of free asphalt increases, and the excess, softer asphalt fills the voids between the aggregates, thus reducing the overall mechanical properties. This non-monotonic variation characteristic can be characterized using an exponential decay function with a peak value. Similarly, the compressive and tensile Poisson’s ratio has a quadratic function relationship with the asphalt content, and there is a minimum point.

Based on this, and combining previous research, quantitative models can be established to describe the compressive–tensile mechanical parameters of cement-stabilized crushed stone and asphalt mixtures as a function of porosity, loading rate, binder content, and temperature. The applicable ranges are shown in Table 8.

#### 3.3.1. Strength

Based on the experimental results and the analysis above, quantitative models for the compressive and tensile strengths of CSM and AM as functions of multiple factors are established, as presented in Equations (6)–(9), respectively:

(6) $$R_{cC}=\left[0.565+\left(0.035+0.454V_{c}-0.021V_{c}^{2}\right)M\right]v^{0.118-0.008M-0.001M^{2}}$$

(7) $$R_{tC}=\left[-0.2+\left(0.277+0.009V_{c}-0.0008V_{c}^{2}\right)M\right]v^{0.031+0.036M-0.006M^{2}}$$

(8) $$R_{cA}=e^{\left(-0.099C^{2}+0.942C+1.207-0.055T\right)}v^{-0.002C+0.2}-4.914+5.364/\left[1+{\left(\frac{V_{c}}{11.857}\right)}^{3.067}\right]$$

(9) $$R_{tA}=e^{\left(-0.145C^{2}+1.42C-1.812-0.061T\right)}v^{-0.001C+0.2}-0.65+0.73/\left[1+{\left(\frac{V_{c}}{12.058}\right)}^{16.226}\right]$$

where *R*_cC_—compressive strength of CSM (MPa). *R*_tC_—tensile strength of CSM (MPa). *R*_cA_—compressive strength of AM (MPa). *R*_tA_—tensile strength of AM (MPa), *M*—cement dosage (%). *C*—asphalt content (%). *T*—test temperature (°C). *v*—loading rate (MPa/s). *V*_c_—air void ratio (%).

The 45° contour map of the compressive and tensile strengths for CSM and AM is presented below to validate the applicability and reliability of the quantitative models.

**Figure 10:**
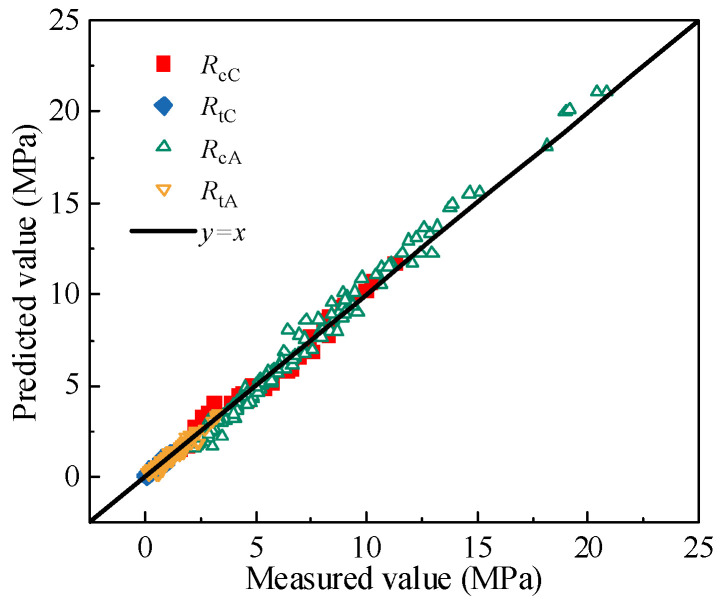
45° contour map of the compressive and tensile strengths.

As illustrated in Figure 10, the predicted and measured values of compressive and tensile strengths for CSM and AM are primarily distributed along both sides of the 45° reference line. The root means square error (RMSE) for the compressive and tensile strengths of CSM is approximately 0.14 and 0.53, respectively, while for the AM, the RMSE values are 0.09 and 1.00. The corresponding R^2^ values are 0.96 and 0.99 for CSM and 0.94 and 0.85 for AM. These results confirm the accuracy and reliability of the proposed models in predicting material strength, offering a valuable reference for evaluating the compressive and tensile strengths of CSM and AM under the influence of multiple factors in practical pavement design.

#### 3.3.2. Modulus

Based on the experimental results and analysis above, quantitative models for the compressive and tensile moduli of CSM and AM are established as functions of multiple factors, as shown in Equations (10)–(13).

(10) $$E_{cC}=\left[\left(-20M+19\right)V_{c}^{2}+\left(497M-372\right)V_{c}+1725M-1290\right]v^{-0.004M^{2}+0.005M+0.15}$$

(11) $$E_{tC}=\left[\left(-14M+12\right)V_{c}^{2}+\left(256M-230\right)V_{c}+1399M-1246\right]v^{-0.0015M^{2}+0.005M+0.17}$$

(12) $$E_{cA}=e^{\left(-0.1C^{2}+0.998C+7.008-0.012CT-0.042T\right)}v^{-0.0005T+0.24}+944-174.4V_{c}$$

(13) $$E_{tA}=e^{\left(-0.109C^{2}+1.052C+6.618-0.001CT-0.052T\right)}v^{-0.00075T+0.27}+431-87.6V_{c}$$

where *E*_cC_—compressive elastic modulus of CSM (MPa). *E*_tC_—tensile elastic modulus of CSM (MPa). *E*_cA_—compressive elastic modulus of AM (MPa). *E*_tA_—tensile elastic modulus of AM (MPa).

To validate the applicability and reliability of the quantitative models for compressive and tensile moduli, the 45° contour maps of compressive and tensile moduli for CSM and AM are presented below.

**Figure 11:**
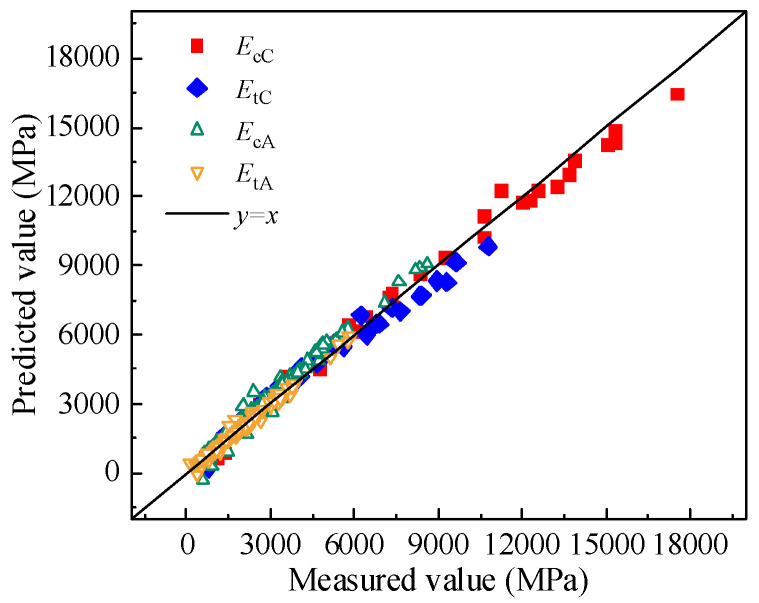
45° contour map of the compressive and tensile moduli.

As illustrated in Figure 11, the predicted and measured values of compressive and tensile moduli for CSM and AM are primarily distributed along both sides of the 45° reference line. The RMSE for the compressive and tensile moduli of CSM is 128.06 and 358.3, respectively, while for AM, the RMSE values are 468.21 and 590.97. The corresponding R^2^ values are 0.99 and 0.96 for CSM and 0.97 and 0.99 for AM. These results confirm the accuracy and reliability of the proposed models in predicting modulus values, providing a valuable reference for evaluating the compressive and tensile moduli of CSM and AM under multi-factor influences in practical pavement design.

#### 3.3.3. Poisson’s Ratio

Based on the experimental results, quantitative models for the compressive and tensile Poisson’s ratios of CSM and AM are developed as functions of multiple influencing factors, as presented in Equations (14)–(17).

(14) $$\mu_{cC}=\left(\left(-0.0003M+0.0031\right){V_{c}}^{2}+\left(0.0057M-0.059\right)V_{c}-0.062M+0.626\right)v^{0.0004{V_{c}}^{2}+\left(-0.0001M-0.009\right)V_{c}-0.012M+0.026}$$

(15) $$\mu_{tC}=\left(\left(-0.0002M+0.0019\right){V_{c}}^{2}+\left(0.003M-0.037\right)V_{c}-0.029M+0.347\right)v^{0.0002{V_{c}}^{2}-0.007V_{c}-0.012M+0.004}$$

(16) $$\mu_{cA}=0.25+\frac{0.508\times 10^{0.05T}}{49.264+10^{0.05T}}+\left(0.0014T-0.003C^{2}+0.026C-0.018\right)10^{-10v}-0.045+0.008V_{c}+\left(3.085\times 10^{-4}\right)V_{c}^{2}$$

(17) $$\mu_{tA}=0.175+\frac{0.147\times 10^{0.05T}}{31.826+10^{0.05T}}+\left(0.0006T-0.004C^{2}+0.038C-0.071\right)10^{-10v}-0.015+0.0003V_{c}+0.0006V_{c}^{2}$$

where *μ*_cC_—compressive Poisson’s ratio of CSM (MPa). *μ*_tC_—tensile Poisson’s ratio of CSM (MPa). *μ*_cA_—compressive Poisson’s ratio of AM (MPa). *μ*_tA_—tensile Poisson’s ratio of AM (MPa).

To validate the applicability and reliability of the quantitative model, 45° contour maps comparing the predicted and measured values of compressive and tensile Poisson’s ratios for CSM and AM are presented below.

**Figure 12:**
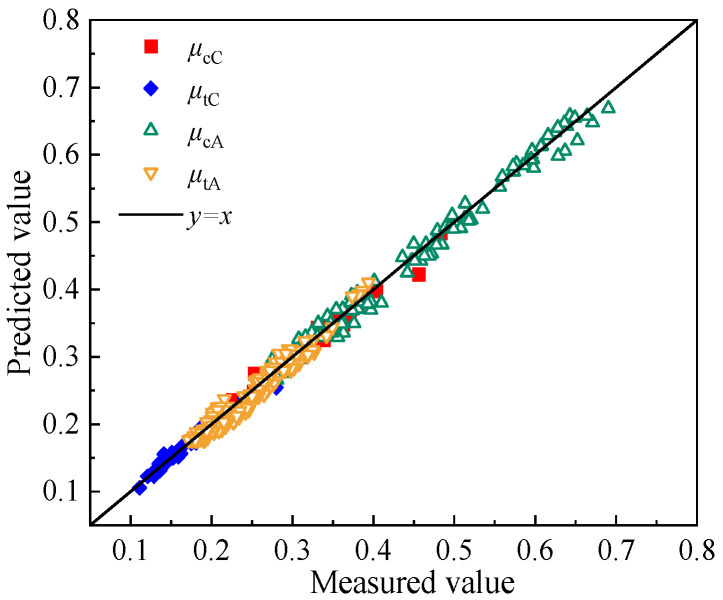
45° contour map of the compressive and tensile Poisson’s ratios.

As shown in Figure 12, the predicted and measured values of compressive and tensile Poisson’s ratios for CSM and AM are closely distributed around the 45° contour line. The RMSE for the compressive and tensile Poisson’s ratios of the two materials is 0.01, 0.02, and 0.02, 0.01, respectively, while the R^2^ values are 0.95, 0.97, and 0.96, 0.98, respectively. These results demonstrate that the models exhibit high accuracy and reliability in predicting Poisson’s ratio, providing a valuable reference for assessing the compressive and tensile Poisson’s ratios of CSM and AM under multi-factor conditions in practical pavement design.

It should be noted that, when the temperature exceeds 30 °C, the asphalt binder shows much stronger viscoelastic and viscoplastic behavior. During loading, the specimen develops non-negligible viscoplastic deformation. As a result, the measured Poisson’s ratio in the uniaxial compression test can exceed 0.5. In this study, however, Poisson’s ratio is calculated from the recoverable strain in the nominal linear-elastic (rebound) stage. The reported value therefore reflects a ratio of recoverable strains under the specific test conditions. A value above 0.5 does not mean that the material’s “true” Poisson’s ratio is greater than 0.5 in the strict sense of isotropic elasticity. Instead, it linearly indicates that, under this temperature and loading condition, the material response has clearly departed from the ideal linear-elastic assumption.

## 4. Study on the Interrelationship of Compressive and Tensile Mechanical Properties for Asphalt Pavement Materials

### 4.1. Relationship Between Strength and Modulus

**Figure 13:**
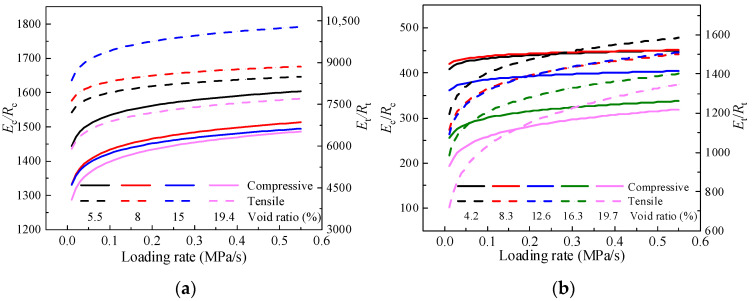
Relationship of *E*_c_/*R*_c_ and *E*_t_/*R*_t_ with influencing factors. (**a**) CSM. (**b**) AM.

Based on the experimental results for the compressive and tensile strengths and moduli of CSM and AM, the variation in the modulus-to-strength ratio under compression (*E*_c_/*R*_c_) and modulus-to-strength ratio under tension (*E*_t_/*R*_t_) with loading rates for the two materials under different gradation types is illustrated in Figure 13.

The variation in *E*_c_/*R*_c_ and *E*_t_/*R*_t_ with loading rates for the two materials is shown in Figure 13. For both CSM and AM, *E*_c_/*R*_c_ and *E*_t_/*R*_t_ increase as the loading rate increases, but the rate of increase gradually decreases. When the loading rate exceeds 0.1 MPa/s, these ratios tend to stabilize. Overall, the influence of loading rate on the modulus and strength ratios is relatively small. For CSM, *E*_c_/*R*_c_ ranges from 1500 to 1600, while *E*_t_/*R*_t_ ranges from 6000 to 10,000. For AM, *E*_c_/*R*_c_ ranges from 200 to 450, and *E*_t_/*R*_t_ ranges from 700 to 1600. Under the same conditions, *E*_t_/*R*_t_ is much greater than *E*_c_/*R*_c_ for all selected gradation types, with differences of more than one order of magnitude for CSM. Therefore, *E*_t_/*R*_t_ is more sensitive to loading rate than *E*_c_/*R*_c_.

Strength and modulus are obtained from two independent tests with different procedures, and the modulus test is relatively more complex. So, the modulus-to-strength ratio provides practical value. Based on the measured variation range of this ratio, the modulus under given conditions can be reasonably estimated using only strength test results, which simplifies the testing process. This approach also provides a useful reference for ensuring compatibility among material parameters during pavement surface course design.

### 4.2. Relationship Between Compressive and Tensile Mechanical Properties

#### 4.2.1. Different Mechanical Characteristics in Compression and Tension of CSM

**Figure 14:**
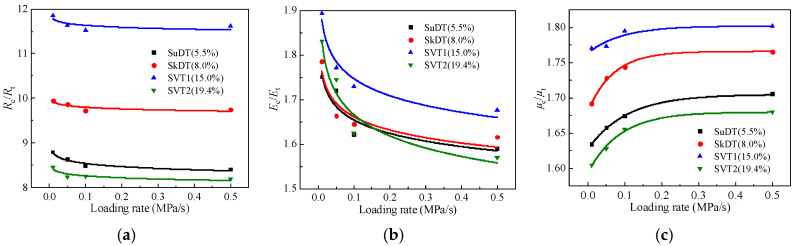
Comparison of compressive and tensile properties for CSM. (**a**) *R*_c_/*R*_t_. (**b**) *E*_c_/*E*_t_. (**c**) *μ*_c_/*μ*_t_.

Based on the test results, the variations in the ratio of compressive and tensile strength (*R*_c_/*R*_t_), the ratio of compressive and tensile modulus (*E*_c_/*E*_t_), and the ratio of compressive and tensile Poisson’s ratio (*μ*_c_/*μ*_t_) with loading rates and air void content are plotted in Figure 14.

Based on Figure 14, the *R*_c_/*R*_t_ and *E*_c_/*E*_t_ of CSM decrease as the loading rate increases, while *μ*_c_/*μ*_t_ increases. All three ratios tend to stabilize once the loading rate reaches 0.1 MPa/s. *E*_c_/*E*_t_ shows the largest variation, about 15%. *R*_c_/*R*_t_ and *μ*_c_/*μ*_t_ are less sensitive to loading rate, with changes within 5%. These results show that, for CSM, the increase in tensile performance with loading rate is generally larger than the increase in compressive performance. This is consistent with the findings in Section 2.1. The reason for this is mainly tied to the load-transfer mechanism. In tension, load transfer relies heavily on bonding in the cement paste and the interfacial transition zone, as well as on crack initiation and growth. A higher loading rate tends to suppress crack propagation and slow damage accumulation. In compression, crack-closure effects, together with aggregate interlock and friction within the skeleton, make the material more stable. As a result, its rate sensitivity is relatively weaker. These observations indicate that the difference in tension and compression of CSM becomes more pronounced as the loading rate increases.

The *R*_c_/*R*_t_ of suspension-dense, skeleton-dense, skeleton-void 1, and skeleton-void 2 CSM are approximately 8.6, 9.8, 11.6, and 8.2, respectively. *E*_c_/*E*_t_ is about 1.6~1.75, 1.6~1.8, 1.65~1.9, and 1.55~1.85, and *μ*_c_/*μ*_t_ is approximately 1.66, 1.73, 1.77, and 1.63, respectively. In addition, the parameter ratios among different gradation types shows that the compressive–tensile parameter ratios are the highest for the skeleton-void 1 gradation and the lowest for the skeleton-void 2 gradation. The reason for this is that, as the internal void content of CSM increases, the tensile parameters change more rapidly than the compressive parameters at the initial stage. When the void content increases to a certain level (around 20%), the compressive mechanical parameters decrease significantly, and the difference between tensile and compressive behavior is reduced.

#### 4.2.2. Different Mechanical Characteristics in Compression and Tension of AM

**Figure 15:**
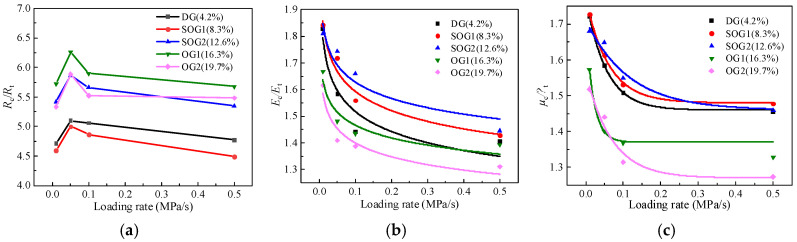
Comparison of compressive and tensile properties for AM. (**a**) *R*_c_/*R*_t_. (**b**) *E*_c_/*E*_t_. (**c**) *μ*_c_/*μ*_t_.

Based on the test results, the variations in the ratio of compressive and tensile strength (*R*_c_/*R*_t_), the ratio of compressive and tensile modulus (*E*_c_/*E*_t_), and the ratio of compressive and tensile Poisson’s ratio (*μ*_c_/*μ*_t_) of AM with loading rates and air void content are plotted in Figure 15.

The results in Figure 15 show that the *R*_c_/*R*_t_ of AM is only weakly affected by loading rate and does not exhibit a clear trend. In contrast, *E*_c_*/E*_t_ and *μ*_c_/*μ*_t_ both decrease as the loading rate increases and tend to stabilize when the loading rate exceeds 0.1 MPa/s. The maximum variation with loading rate is about 30% for *E*_c_*/E*_t_ and about 20% for *μ*_c_/*μ*_t_. This behavior is mainly because a higher loading rate shortens the loading duration. The viscous flow and stress relaxation of the asphalt mastic do not have enough time to fully develop. As a result, the mixture shows higher instantaneous stiffness and load-carrying capacity. In tension, crack growth and interfacial debonding are more pronounced. The response depends more on the cohesion of the asphalt mastic and the bonding at the interfaces. These mechanisms are more sensitive to loading rate. Therefore, the tensile performance usually improves more than the compressive performance. This reduces the difference in tension and compression of AM as the loading rate increases.

By comparing different gradation types, it is found that the compressive–tensile parameter ratios are largest for the semi-open-graded mixtures and smallest for the open-graded 2 mixture. This is mainly because, when the air void content increases within about 10%, the tensile parameters change more rapidly than the compressive parameters at the initial stage. As the air void content continues to increase to around 20%, both compressive and tensile mechanical parameters decrease to relatively low levels, and the difference between them is reduced. Specifically, when the air void content increases from 4.0% to 20%, the ranges of *R*_c_/*R*_t_, *E*_c_*/E*_t_, and *μ*_c_/*μ*_t_ are approximately 5~6, 1~2, and 1~2, respectively. When the loading rate increases from 0.01 MPa/s to 0.5 MPa/s, the ranges of *R*_c_/*R*_t_, *E*_c_*/E*_t_, and *μ*_c_/*μ*_t_ are about 4.4~6.3, 1.3~1.8, and 1.2~1.7, respectively.

### 4.3. Verification of the Basic Assumption for Bi-Modulus Theory

In bi-modulus theory, the fundamental mechanical parameters of materials should satisfy the following basic assumption:

(18) $$E_{c}/\mu_{c}=E_{t}/\mu_{t}$$

(19) $$Dev=\left(E_{c}/\mu_{c}-E_{t}/\mu_{t}\right)/\left(E_{t}/\mu_{t}\right)$$

**Figure 16:**
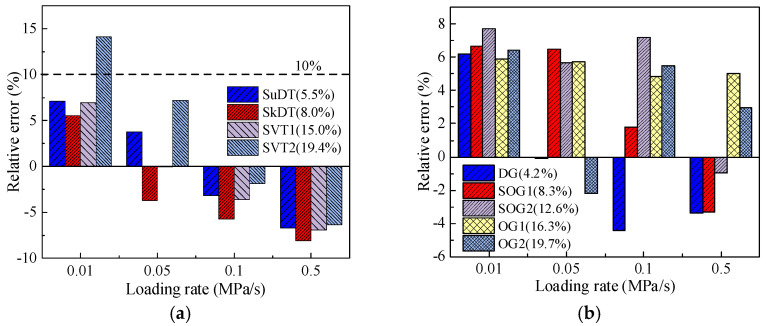
Deviation analysis diagram of *E*_c/t_/*μ*_c/t_. (**a**) CSM. (**b**) AM.

To verify the applicability of the bi-modulus theory in asphalt pavement materials, this study calculates the ratios of compressive-to-tensile modulus and Poisson’s ratio based on laboratory-measured mechanical parameters of CSM and AM under various influencing factors. The deviation (*Dev*) between these ratios is computed using Equation (19), with the results presented in Figure 16.

As shown in Figure 16, the deviations of both CSM and AM under different void ratios and loading rates generally remain within 10%. Only under the condition of 0.01 MPa/s and a 19.4% void ratio does CSM deviation exceed 10%. This anomaly may be attributed to insufficient specimen deformation at extremely low loading rates or non-uniform mixing during specimen preparation.

Furthermore, based on the fundamental bi-modulus assumption (*E*_c_/*μ*_c_ = *E*_t_/*μ*_t_), one mechanical parameter was calculated and compared with experimental measurements to further validate the applicability of bi-modulus theory for asphalt pavement materials. Taking direct tensile modulus as an example, the 45° equivalence line plot between calculated and measured results is presented below.

**Figure 17:**
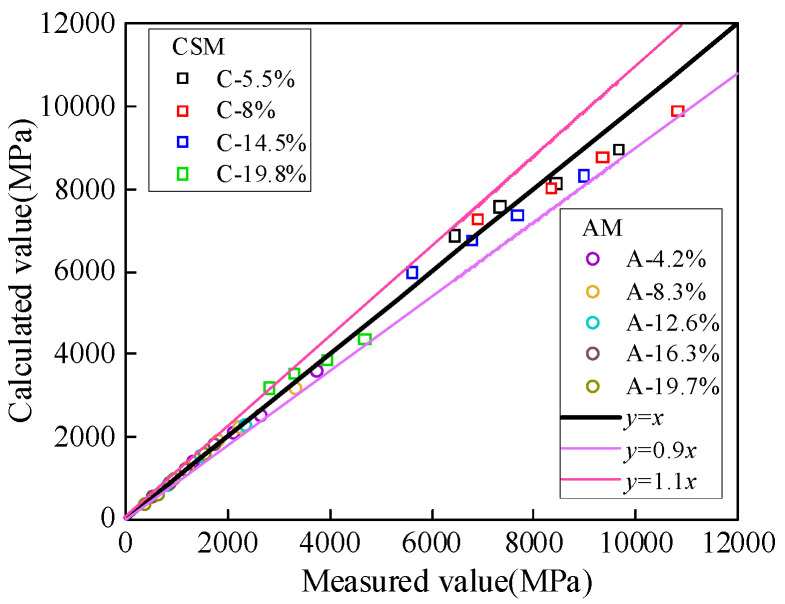
45° equivalence line plot between calculated and measured results.

As illustrated in Figure 17, the calculated results and measured values are distributed on both sides of the equivalence line, with maximum relative errors generally within 10%. Therefore, by synthesizing the findings from Figure 16, it can be concluded that the tensile and compressive mechanical parameters of both CSM and AM under varying void ratios and loading rates fundamentally satisfy the basic assumption of bi-modulus theory (*E*_c_/*μ*_c_ = *E*_t_/*μ*_t_).

## 5. Conclusions

The tensile and compressive mechanical behavior of typical pavement materials is influenced by multiple factors, including material composition, environmental conditions, and loading characteristics. This makes parameter selection in current pavement structural design less reliable. To address this issue, this study investigates the nonlinear tensile and compressive responses of CSM and AM under different gradation types and loading rates. And a quantitative value model was established to describe the variation in mechanical parameters of typical asphalt pavement materials, which vary in air void ratio, loading rate, binder content, and temperature. The main conclusions are as follows:

(1) The skeleton-dense cement-stabilized macadam shows the highest strength and modulus and the lowest Poisson’s ratios. For semi-open graded asphalt mixtures, the tensile and compressive mechanical parameters are strongly affected by air void content, with variations of up to 57% as the air voids increase from 8% to 16%. The rates of decrease in strength and modulus and the rate of increase in Poisson’s ratios are all high. For both materials, the tensile and compressive parameters follow a power-law relationship with loading rate and tend to stabilize when the loading rate reaches 0.1 MPa/s. On this basis, a multi-factor quantitative model was developed for the tensile and compressive strength, elastic modulus, and Poisson’s ratio of typical asphalt pavement materials used in surface and base layers.

(2) In terms of the significance of influencing factors, gradation type has a much stronger effect on tensile and compressive parameters than loading rate. Among the individual parameters, the elastic modulus is most affected by both gradation and loading rate, with its maximum variation exceeding threefold. Regarding the tensile and compressive characteristics, tensile parameters are more sensitive than compressive ones to changes in these factors.

(3) With respect to the materials and scope investigated in this study, the skeleton-dense CSM exhibits the best overall mechanical performance. Under this condition, *R*_c_/*R*_t_ reaches up to 10, while *E*_c_/*E*_t_ and *μ*_c_/*μ*_t_ are approximately 1.7. *E*_c_/*R*_c_ and *E*_t_/*R*_t_ can be 1450 and 9000, respectively. For AM, *R*_c_/*R*_t_ can reach 5, with *E*_c_/*E*_t_ and *μ*_c_/*μ*_t_ being around 1.5. *E*_c_/*R*_c_ and *E*_t_/*R*_t_ range from 250 to 450 and from 700 to 1600, respectively.

(4) CSM and AM exhibit significantly nonlinear behavior in compression and tension. Their stress–strain behavior under tension and compression conforms to the bilinear characteristics of the bi-modulus theory. The deviation between the ratios of compressive and tensile modulus to Poisson’s ratio for both materials under different gradation types and loading rates is generally within 10%. This agrees with the assumption that *E*_c_/*μ*_c_ = *E*_t_/*μ*_t_ and supports the applicability of Ambartsumyan’s bi-modulus constitutive theory to typical pavement materials.

## Figures and Tables

**Table 1:** Summary of cement technical indexes.

Test Content		Test Value	Technical Requirements
Fineness test (80 μm sieve analysis method)		3.2	≤10%
Setting time (min)	Initial setting	245	≥180
	Final setting	425	≥360
Stability (Reye clamp method) (mm)		3.3	≤5
Cement mortar strength (MPa)	3 d flexural tensile strength	4.0	≥2.5
	28 d flexural tensile strength	≥5.5	―
	3 d compressive strength	18.4	≥10
	28 d compressive strength	≥32.5	―

**Table 2:** Test results of SBS-modified asphalt.

Test Content	Test Value	Technical Requirements
Penetration 25 °C, 100 g, 5 s (0.1 mm)	48.5	30–60
Ductility 5 cm/min, 5 °C (cm)	26.3	≥20
Softening point (°C)	78.0	≥60
Kinematic viscosity 135 °C (Pa·s)	1.6	≤3
Elastic recovery 25 °C (%)	80	≥75
Solubility (%)	99.5	≥99
Flashpoint (°C)	255	≥230

**Table 3:** Test results of mechanical properties of aggregate.

Test Content	Test Value	Technical Requirements
Crushing value (%)	14.5	≤28
Polished stone value (BPN)	62.3	≥45
Abrasion value (%)	12.3	≤30

**Table 4:** Grading design of CSM.

Gradation Type	Screen Size (mm)											
	26.50	19.00	16.00	13.20	9.50	4.75	2.36	1.18	0.60	0.30	0.15	0.075
Suspension-dense type (SuDT)	100	92.5	85.5	77.0	62.7	34.4	21.2	17.0	11.5	8.2	5.1	3.6
Skeleton-dense type (SkDT)	100	84	76	68	56	39	25	16	10	8	5	3
Skeleton-void type 1 (SVT1)	100	80	70	58	46	28	20	10	6	3	2	1
Skeleton-void type 2 (SVT2)	100	80	68	52	38	18	12	5	3	1	0	0

**Table 5:** Grading design of AM.

Gradation Type	Screen Size (mm)										
	19.00	16.00	13.20	9.50	4.75	2.36	1.18	0.60	0.30	0.15	0.075
Dense gradation (DG)	100	95.4	86.5	70.5	45.0	30.2	23.0	15.4	10.7	7.1	5.3
Semi-open gradation 1 (SOG1)	100	92	80	62	32	22	15	10	7	5	3
Semi-open gradation 2 (SOG2)	100	93	74	57	28	17	12	8	6.5	5	3
Open gradation 1 (OG1)	100	96	80	54	21.6	16	12	10	7.3	5.3	3
Open gradation 2 (OG2)	100	96	76	48	16	13.5	10	7.5	5.3	4.8	3

**Table 6:** Maximum dry density and optimum moisture content of CSM.

Gradation Type	Void Ratio (%)	Maximum Dry Density *ρ*_dmax_ (g/cm^3^)	Optimum Moisture Content ω_0_ (%)
SuDT	5.50	2.30	4.64
SkDT	8.00	2.32	4.55
SVT1	15.00	2.19	4.35
SVT2	19.40	2.12	4.10

**Table 7:** Marshall test results of AM.

Gradation Type	Maximum Theoretical Density (g/cm^3^)	Gross Bulk Density (g/cm^3^)	Void Ratio (%)	Asphalt Saturation (%)	Stability (kN)	Flow Value (mm)
DG	2.422	2.322	4.2	70.7	15.4	2.3
SOG1	2.431	2.227	8.3	74.3	13.6	2.9
SOG2	2.511	2.193	12.6	77.6	12.1	4.2
OG1	2.563	2.131	16.3	80.4	10.9	5.6
OG2	2.621	2.102	19.7	82.5	8.7	6.2

**Table 8:** Influencing factors and their research level.

Influencing Factors	Cement-Stabilized Macadam	Asphalt Mixture
Air void ratio (%)	5.5, 8.0, 15.0, 19.4	4.2, 8.3, 12.6, 16.3, 19.7
Loading rate (MPa/s)	0.01, 0.05, 0.1, 0.5	0.01, 0.05, 0.1, 0.5
Binder content (%)	1, 2, 3, 4, 5	3.5, 4, 4.5, 5, 5.5
Temperature (℃)	5, 15, 20, 30, 40	5, 15, 20, 30, 40

## Data Availability

The original contributions presented in this study are included in the article. Further inquiries can be directed to the corresponding authors.

## References

[1] Tan Z.F., Yang B., Leng Z., Jelagin D., Cao P., Li R., Zou F. (2023). Multiscale characterization and modeling of aggregate contact effects on asphalt concrete’s tension-compression asymmetry. Mater. Des..

[2] Tong J.S., Shen K.R., Ma T., Zhang J. (2022). Characterizing the tension-compression asymmetric viscoelasticity of asphalt mixture using the uniaxial tension and compression test. Constr. Build. Mater..

[3] Norouzi A., Kim D., Kim Y.R. (2016). Numerical evaluation of pavement design parameters for the fatigue cracking and rutting performance of asphalt pavements. Mater. Struct..

[4] Tavares A.L.D., Furlan A.P., Fabbri G.T.P., Pedreira J.U. (2025). Soil-aggregate-cement mixtures for base pavement layers: A strength and stiffness characterization. Transp. Geotech..

[5] Barra B., Neckel G., Momm L., Guerrero Y., Mikowski A., Staub de Melo J.V., Nguyen M.-L., Hughes G.B. (2020). Design parameters and associated quantitative damage analyses of an asphalt concrete airfield runway. Constr. Build. Mater..

[6] Wang N.H., Zhang T.Z. (2011). Experimental study on material composition and indoor performance of permeable cement stabilized macadam base mixture. J. China Foreign Highw..

[7] Zhou Z.G., Li S.S., Liu X.G., Yu W.-S., Yang Z.-F. (2016). Evaluation of anti-erosion performance of SMA mixtures. China J. Highw. Transp..

[8] Liu Z.N., Sun L.J., Li J.H., Liu L. (2022). Effect of key design parameters on high temperature performance of asphalt mixtures. Constr. Build. Mater..

[9] Mansour T.N., Putman B.J. (2013). Influence of aggregate gradation on the performance properties of porous asphalt mixtures. J. Mater. Civ. Eng..

[10] Zhou Z.G., Liu X.G., Luo S.P., Sha X. (2016). Effect of water intrusion on performance of asphalt mixture. J. Cent. South Univ. (Nat. Sci.).

[11] Zhou H., Sha A.M. (2012). Analysis on the influences of material composition on semi-rigid base fatigue property. J. Wuhan Univ. Technol..

[12] Jiang Y.J., Li M.J., Zhang J.J., Wang S. (2010). Influence factors of strength properties of cement stabilization of crushed aggregate. J. Chang’an Univ. (Nat. Sci. Ed.).

[13] Li M.L., Xie J., Wu S.P., Chen J. (2023). Study on the influence mechanism of recycled concrete aggregate on strength of asphalt mixtures. Constr. Build. Mater..

[14] Qabur A., Baaj H., El-Hakim M. (2025). A laboratory study on enhancing asphalt mixture properties through dry mixing with high-dose multilayer plastic packaging pellet additives. J. Mater. Civ. Eng..

[15] González-Martínez M.A., Gómez-Soberón J.M., Leal-Castañeda E.J. (2025). Permeable concrete with recycled aggregates: Study of its mechanical and microstructural properties. Materials.

[16] Behnke R., Kaliske M., Schuck B., Stein M., Alber S., Ressel W., Wellner F., Leischner S., Canon Falla G., Eckstein L. (2023). From the material behaviour to the thermo-mechanical long-term response of asphalt pavements and the alteration of surface drainage due to rutting: A sensitivity study. Int. J. Pavement Eng..

[17] Pan Q.X., Hu J., Zheng J.L., Lv S., Zhang J., Wen P., Song X. (2024). Poisson’s ratio testing method and multi-factor influence quantification model of asphalt pavement materials. Constr. Build. Mater..

[18] Timoshenko S. (1941). Strength of Materials, Part II: Advanced Theory and Problems.

[19] Ambartsumyan S.A. (1986). Elasticity Theory of Different Modulus.

[20] Lv S.T., Li Y.P., Liu C.C., Zheng J.-L. (2017). Synchronous testing method for tensile and compressive moduli of asphalt mixture based on splitting test. China J. Highw. Transp..

[21] Quang T.N., Di Benedetto H., Sauzeat C., Nguyen M.L., Hoang T.T.N. (2017). 3D complex modulus tests on bituminous mixture with sinusoidal loadings in tension and/or compression. Mater. Struct..

[22] Tan Z.F., Li H., Leng Z., Jelagin D., Cao P., Du C., Yin B. (2024). Constitutive modelling and systematic evaluation of asphalt concrete’s viscoelastic tension-compression asymmetry effect on pavement performance. Int. J. Pavement Eng..

[23] Yao W.J., Ye Z.M. (2004). The analytical solution of bending beams transverse force based on different modulus. Appl. Math. Mech..

[24] Wu X., Yang L.J., Huang C. (2010). Large deflection bending calculation and analysis of bi-modulus rectangular plate. Eng. Mech..

[25] Zhang Q.S., Zheng J.L. (1992). A calculation method for rigid pavement considering the difference of tensile and compressive modulus. J. Changsha Commun. Inst..

[26] Pan Q.X., Zhang T.S., Wei Y.F., Zhen X., Sun J., Song X., Jiang Y., Jiang S., Hu J. (2024). Mechanical analysis of typical asphalt pavement based on bi-modulus theory. J. Transp. Sci. Eng..

[27] (2020). Testing Methods of Cement and Concrete for Highway Engineering.

[28] (2025). Standard Test Methods of Asphalt and Asphalt Mixture for Highway Engineering.

[29] (2024). Test Methods of Aggregates for Highway Engineering.

[30] (2024). Test Methods of Materials Stabilized with Inorganic Binders for Highway Engineering.

[31] Pan Q.X., Zhou H.D., Zheng C.C., Liu H., Lv S., Song X., Zhang J., Yang B., Yue D. (2024). Matching relationships for mechanical parameters of asphalt pavement materials considering difference in tension and compression. Int. J. Pavement Res. Technol..

[32] Huang T., Wang Z.Y., Dong H.W., Qin H., Liu H., Cao Z. (2024). Time-Temperature-Stress Equivalent Characteristics and Nonlinear Viscoelastic Model of Asphalt Mixture under Triaxial Compressive Stress State. J. Mater. Civ. Eng..

[33] Huang T., Li M., Yang Y., Xie J., Liu H., Yao H., Xu Q., Qi S. (2020). Unified Strength Models of an Asphalt Mixture under Different Temperatures and Three-Dimensional Stresses. J. Mater. Civ. Eng..

[34] Gong X.B., Huang X., Qian G.P., Liu H. (2023). Effect of thickness on the behavior of aggregate-asphalt-aggregate film subjected to dynamic shearing and uniaxial tension. Constr. Build. Mater..

[35] Pan Q.X., Jiang S.J., Lv S.T., Zheng J. (2024). Mechanical characterization of tensile and compressive differences in asphalt mixtures in multifactor coupling. J. Tongji Univ. (Nat. Sci.).

